# Differentiating the Effects of Fathers’ and Mothers’ Parenting Styles on Academic and Emotional Outcomes among Male and Female Vietnamese Students

**DOI:** 10.11621/pir.2025.0403

**Published:** 2025-12-01

**Authors:** Pham Quang Dao, Le Duy Hung

**Affiliations:** a Ural Federal University named after the first President of Russia B. N. Yeltsin, Yekaterinburg, Russia; b Ho Chi Minh City University of Education, Vietnam

**Keywords:** parenting styles, academic outcomes, emotional outcomes, social comparison, gender differences

## Abstract

**Background.:**

Parenting styles shape children’s academic performance and psychological well-being, yet previous research has not distinguished the separate effects of fathers and mothers on boys and girls, nor has it examined the mediating role of social comparison in these relationships.

**Objective.:**

To examine gender-specific differences in the effects of fathers’ and mothers’ parenting styles and explore the mediating role of social comparison among Vietnamese high school students.

**Design.:**

A sample of Vietnamese high school students (N = 661) completed self-report measures assessing perceived parenting styles, social comparison, academic performance, and psychological distress levels.

**Results.:**

The findings revealed that while fathers’ authoritative and authoritarian styles were not signiicantly associated with boys’ academic performance, authoritarian parenting was linked to lower academic outcomes among girls. Fathers’ permissive style showed a positive association with girls’ academic performance but was also related to higher distress levels across both genders. For mothers, the authoritarian style appeared more detrimental to girls’ academic outcomes than to boys’, whereas the authoritative style was associated with lower distress only among girls. Regarding indirect effects, social comparison played a signiicant mediating role in several relationships, particularly for mothers’ parenting. Specifically, mothers’ authoritarian style was related to higher academic performance and greater distress through social comparison in both genders. Conversely, mothers’ authoritative style improved boys’ academic performance via social comparison, whereas fathers’ permissive style was associated with improved academic performance among girls and increased distress across genders through the same mechanism.

**Conclusion.:**

Our findings highlight the need for gender-sensitive parenting interventions that consider the distinct roles of fathers and mothers in shaping academic and psychological outcomes. Additionally, promoting healthy social comparison strategies in schools may help mitigate distress and enhance students’ academic motivation.

## Introduction

In developmental psychology, research on parenting styles has long been a central focus due to its profound influence on children’s development. Prior studies have affirmed that authoritative parenting contributes to reducing symptoms of depression and anxiety while enhancing children’s self-confidence (Milevsky et al., 2007; Romero-Acosta et al., 2021). Concurrently, studies further indicate that parenting styles not only affect children’s self-regulation and self-awareness, but also directly impact academic performance by fostering essential social skills (Azman et al., 2021; Szkody et al., 2021; Wilder, 2014). Parenting behaviors rooted in acceptance and supportive autonomy not only improve academic outcomes, but also facilitate emotional self-regulation and overall mental well-being (Bibi et al., 2022; Peng et al., 2021).

However, despite substantial evidence of the role of parenting styles in mental health and academic success, inconsistencies remain within the existing literature. Gawas (2021) found that parenting styles, whether authoritative, authoritarian, or permissive, have no impact on students’ academic performance. Stavrulaki et al. (2021) reported that authoritative parenting styles have no impact on students’ grade point averages. On the other hand, some studies have shown that while authoritative parenting is generally associated with higher academic performance, its effects can be moderated by factors such as intrinsic motivation, parental education levels, and socioeconomic status (Azman et al., 2021; Hassan et al., 2022). Moreover, prior studies suggested that a combination of an authoritarian maternal style and a permissive paternal style negatively impacts internalizing symptoms in boys while demonstrating different patterns of influence on girls’ developmental outcomes (Braza et al., 2015; Yaffe, 2023). These variations highlight the necessity of considering both child gender and familial context when analyzing the impact of parenting styles.

A large-scale study in Taiwan found that while parental support universally predicted better mental health and academic grades for both boys and girls, the efect of parental control was gender-specific (Yu & Ho, 2023). Paternal control was found to be detrimental to the academic performance of both sexes, yet maternal control was positively associated with academic performance only for sons. These findings support the idea that the same parenting style from diferent parents may have diferent effects on academic outcomes based on the gender of adolescents. This interactional complexity is similarly evident in studies focusing on psychological distress, such as anxiety and depression. A longitudinal study from the Philippines has shown that authoritarian and neglectful mothering styles predicted higher depressive symptoms in daughters three years later, but not in sons (Hock et al., 2018). In a sample of Chinese adolescents, mothers were perceived as warmer and more demanding than fathers; however, fathers’ academic pressure exhibited a stronger association with adolescents’ anxiety and depression (Quach et al., 2015). These results highlight gender differences in the relationship between parenting styles and children’s mental health and academic outcomes.

Beyond the direct relationship between parenting styles and children’s outcomes, several studies reveal mediating mechanisms in this relationship. Frameworks such as Self-Determination Th eory (Ryan & Deci, 2000) emphasize the roles of needs for autonomy, competence, and relatedness in shaping motivation and behavior. Costa et al. (2019) found that adolescents’ fulfillment of these needs mediated the associations between six parenting dimensions and psychological adjustment, with supportive parenting enhancing need satisfaction and positive outcomes, while controlling parenting undermined these needs and led to poorer adjustment. Similarly, longitudinal evidence shows that parental behaviors supporting or thwarting autonomy and relatedness operate as mediating pathways between parental functioning and child outcomes, as demonstrated in studies examining maternal substance use (Guo & Slesnick, 2018) and parental stress effects on children’s social competence (Devina & Hendrawan, 2023). Furthermore, cross-sectional and panel studies have established that basic need satisfaction mediates the relationship between parental autonomy support versus psychological control and various child outcomes, including alexithymia (Barberis et al., 2022) and school adjustment through sequential mediation involving autonomy and academic stress (Yeon & Choi, 2022). However, these studies tend to focus on internal mechanisms and may overlook the social-cognitive processes through which adolescents interpret and evaluate parental expectations. Scholars have increasingly recognized that the meaning and impact of parenting behaviors are filtered through adolescents’ perceptions of their own standing relative to peers and the expectations they perceive in the social environment. Liu et al. (2025) showed that parental emphasis on achievement and social comparison influences adolescents’ self-esteem indirectly through comparisons with peers. This shift highlights the potential importance of self-evaluative and comparative processes that could mediate the link between parenting and children’s outcomes. In this context, social comparison could become a potential mediating variable in this relationship.

According to Social Comparison Theory (Festinger, 1957), individuals have a tendency to evaluate themselves by comparison with others in order to determine personal competence, social value, and group status. In East Asian societies, social comparison is ot en employed by parents as an educational and behavioral control strategy, with the expectation that children will strive harder to meet the standards demonstrated by others (Liu et al., 2025). Many Vietnamese parents use peer comparison as a way to express afection and a desire for their children to excel beyond others (Dang & Nguyen, 2025). However, when comparison becomes a persistent management tool without accompanying emotional support, children may experience psychological pressure and may develop negative symptoms such as anxiety or depression (Lôc et al., 2024; Nguyêt, 2010). In this context, social comparison is not merely an individual phenomenon, but becomes a social structure that inluences the entire trajectory of a child’s psychological development.

### Authoritative Parenting in the Vietnamese Context and the Mediating Role of Social Comparison

Authoritative parenting is characterized by a balanced combination of reasonable control, warmth, respect for autonomy, and open two-way communication. In Western cultures, this parenting style is often considered ideal, as it fosters self-confidence, self-regulation, and high academic achievement in children. However, in Vietnam, where collectivist values and academic performance are culturally emphasized, the effects of this parenting style may be different. In Vietnamese families where love is combined with reasonable discipline, children tend to exhibit higher academic motivation, active engagement in learning activities, and signiicantly reduced symptoms of depression and anxiety (La et al., 2020; Thao, 2025). Parents with this style often encourage emotional expression, guide learning based on personal needs, and help set appropriate goals, thereby fostering greater self-regulation and academic motivation (Agbaria et al., 2021; Thao, 2025). Nonetheless, in the Vietnamese context, the openness characteristic of authoritative parenting may be constrained by specific cultural conditions (Lôc et al., 2024). In a collectivist society like Vietnam, where academic achievement serves as a primary indicator of personal and familial worth, children may still encounter intense social comparison pressures outside the home. Chung and Mallery (1999) noted that students in collectivist cultures tend to engage in social comparison more frequently than those in individualistic societies. Vietnam’s educational system, with features such as class rankings, public awards, and absolute grading standards, continuously compels students to evaluate their self-worth through performance relative to others. Furthermore, as personal value in Vietnamese society is often tied to the ability “to keep up with peers” (*bang ban bang* è), the emotional and psychological support from authoritative parents can be undermined if the child is immersed in a highly competitive academic or community environment that persistently reinforces academic performance-based comparison. The dissonance between the flexible expectations at home and the rigid standards outside creates a dual pressure, pulling children between the desire for intrinsic self-affirmation and the need for external validation based on social norms. This tension may render the authoritative parenting style less efective in practice in Vietnamese society.

### Authoritarian Parenting in the Vietnamese Context and the Mediating Role of Social Comparison

Authoritarian parenting is characterized by high levels of control, strict demands for obedience, and limited emotional communication between parents and children. In Western countries, this style is generally associated with negative outcomes, such as lower academic performance and elevated risks of anxiety and depression (Goros-tiaga et al., 2019; Pinquart, 2016). However, in East Asian contexts such as Vietnam, this parenting style does not uniformly predict negative outcomes and may exert dual efects. In Vietnam, many parents continue to employ strict parenting methods as a way to ensure their children’s academic success (Dang & Nguyen, 2025). From an early age, children are oten placed within rigid routines such as extended study hours, high expectations for academic achievement, and harsh consequences for failure. Within this framework, social comparison is frequently utilized by parents not only as a behavioral correction tool but also as a motivational strategy. This suggests that, within the Vietnamese manifestation of authoritarian parenting, social comparison is not merely a spontaneous behavior initiated by children, but more a parent-driven, structurally reinforced control mechanism.

Drawing on Social Comparison Theory (Festinger, 1957), it becomes evident that when social comparison is imposed as a mandated evaluative norm, it can become a significant source of psychological distress. Children are pressured to assess themselves against external benchmarks of success, leading to a disconnection between intrinsic values and external validation. Moreover, when parental control is accompanied by negative comparative feedback, children’s self-esteem may become unstable and overly reliant on external recognition and social approval. Despite these risks, it is important to acknowledge that some students in such environments still achieve high academic performance (Watabe & Hibbard, 2014). Nevertheless, this positive outcome often come at the cost of emotional distress and impaired self-regulation. In the Vietnamese context, increasing evidence points to alarming rates of suicide, suicidal ideation, and self-harming behaviors among students, many of which are linked to extreme academic pressure (Giang et al., 2023; Mai, 2024). This underscores the notion that the academic beneits of authoritarian parenting may be attained at a signiicant psychological cost, especially when social comparison mechanisms are not moderated by emotional support from parents.

### Permissive Parenting in the Vietnamese Context and the Mediating Role of Social Comparison

Permissive parenting is characterized by low levels of control, a lenient approach to setting boundaries, and a tendency to avoid conflict or strict disciplinary strategies. This parenting style is commonly associated with poor academic outcomes, low self-regulation, and elevated psychological distress (Goagoses et al., 2023; Timpano et al., 2015; Yang & Zhao, 2020). According to Social Comparison Theory (Festinger, 1957), when strong internal standards are absent, individuals tend to rely on external cues to evaluate their self-worth and social position. In the case of children raised in permissive households, the development of robust internal standards is oten hindered, making them more vulnerable to external influences when forming self-evaluations. Although children of permissive parents may not experience direct academic pressure from their families, they are nonetheless frequently exposed to external pressures and peer comparison, especially in competitive school environments. This phenomenon is particularly salient in Vietnam, where the educational system emphasizes academic achievement, class rankings, and expectations from teachers, schools, and the wider community.

Several empirical studies conducted in Vietnam have indirectly conirmed these associations. A study in Hue found that students with lower academic performance tended to report higher levels of school-related stress, even in the absence of strong parental pressure (Thao, 2025). This indicates that environmental academic stress remains a dominant force, with signiicant implications for students’ psychological well-being. Moreover, research by La et al. (2020) in Viet Nam revealed that adolescents with parents who exhibited low guidance but high emotional indulgence were nearly twice as likely to experience psychological diiculties compared to those raised by authoritative parents. Th ese findings suggest that in high-demand educational contexts such as Vietnam, permissive parenting does not shield children from social pressure. Instead, it may undermine their adaptive capacity and increase their susceptibility to psychological vulnerability. In the absence of parental direction, children may increasingly depend on social comparison as a compensatory mechanism to evaluate their status and performance. This reliance on external benchmarks can generate chronic stress, anxiety, and inferiority, particularly when children perceive themselves as failing to meet societal or peer-related expectations (Gilbert et al., 2009). Thus, rather than serving as a buffer, permissive parenting may inadvertently exacerbate children’s exposure to harmful comparison-based evaluations in academically competitive environments.

### Gendered Parenting Roles in Vietnam

Parenting roles tend to be divided along gender lines, with fathers and mothers playing distinct but complementary roles in Vietnamese families. Fathers are often seen as authoritative igures responsible for setting long-term goals, enforcing discipline, and making strategic decisions regarding the children’s education and future (Düng, 2007). Mothers, by contrast, provide direct emotional support and caregiving in daily life (Hiên, 2023; Linh, 2019). This division reflects enduring cultural norms such as “strict father, gentle mother” (*cha nghiêm, me tù*), which shapes the way children interpret and respond to parental behavior. In modern Vietnamese educational settings, where academic competitiveness and social comparison are prominent, the parenting dynamic becomes more complex. Children lacking clear academic guidance from either parent often turn to peers as primary reference points. In such cases, both boys and girls are exposed to the pressure of social comparison, albeit in gendered ways. Girls, being more sensitive to peer evaluation (Guyer et al., 2014), may experience greater emotional distress when perceiving themselves as underperform-ing. Boys, while less expressive, are not immune to the efects of being compared with others (Liu et al., 2025; Tian et al., 2017). These findings suggest that parenting styles and child gender may be associated with variations in how social comparison processes relate to psychological distress and academic outcomes in Vietnam.

**Figure 1.**
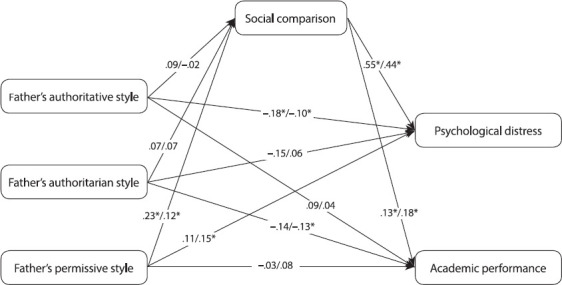
Fathers’ parenting roles in shaping children’s academic outcomes and psychological distress (male/female).

Note. *p < .05.

**Figure 2.**
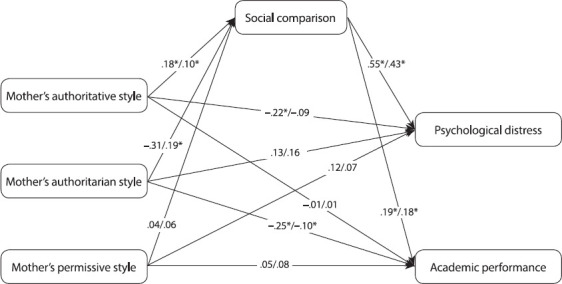
Mothers’ parenting roles in shaping children’s academic outcomes and psychological distress (male/female)

Note. *p < .05.

However, to the best of our knowledge, there remains a notable lack of empirical evidence regarding the role of social comparison in explaining the associations between parenting styles and children’s mental health and academic achievement across genders. This gap limits our understanding of how boys and girls may differentially experience and respond to paternal and maternal parenting practices through social comparison. Therefore, the present study was designed to analyze the distinct impact of both fathers’ and mothers’ parenting style on boys’ and girls’ academic performance and psychological distress. Second, it investigates the mediating role of social comparison in explaining how parenting styles impact these outcomes (*Figure 1 and Figure 2*). Such an investigation could help address the inconsistencies in the existing literature and provide empirical evidence to clarify and validate the role of social comparison in the relationship between parenting styles, academic achievement, and children’s mental health.

## Methods

### Participants

A cross-sectional study was designed to investigate these relationships among Vietnamese high school students. Participants were recruited from both public and private high schools located in urban and suburban areas in southern Vietnam. Students from diferent grades and classes were included to ensure sample diversity. However, the sample was limited to southern Vietnam, which may restrict the gen-eralizability of the findings to other regions. Ethical approval was obtained from the school administration before data collection. All procedures complied with ethical guidelines for research involving minors. The questionnaire was then distributed to classrooms via a Google Form link, with clear instructions on how to complete the survey and an explanation of participants’ right to voluntarily participate or withdraw without providing a reason. Students provided informed consent and participated voluntarily and anonymously. A total of 661 students participated and completed the questionnaire. All responses were valid and included in the analysis, consisting of 246 male and 415 female students. Further details of the participants are presented in *Table 1*.

### Procedure

#### Questionnaires

The 30-item version of the Parenting Style Questionnaire (Buri, 1991) was used to assess students’ perceived parenting styles for both their father and mother. Participants responded to 60 identical items—30 for their father and 30 for their mother. This scale measures three key parenting styles: authoritative, authoritarian, and permissive. It was adapted for high school students to ensure appropriateness. Participants rated items on a 6-point Likert scale ranging from 1 (Never) to 6 (Always). Scores for each parenting style relect the extent to which students perceive their parents as frequently using that style. The original English version was translated into Vietnamese following a forward-backward translation procedure (Brislin, 1986). The father’s authoritative style showed excellent internal consistency (Cronbach’s alpha (α) = .96), strong composite reliability (CR) = .96, and good convergent validity (Average Variance Extracted (AVE) = .67). Likewise, the father’s authoritarian style exhibited high reliability (α = .94, CR = .95) and satisfactory convergent validity (AVE = .60). The father’s permissive style showed acceptable levels (α = .66, CR = .80, AVE = .50), meeting the .50 threshold for AVE, thus supporting its inclusion despite slightly lower internal consistency (Fornell & Larcker, 1981). For maternal parenting, the mother’s authoritative style was highly reliable (α = .96, CR = .96, AVE = .66), and the mother’s authoritarian style demonstrated strong psychometric quality (α = .95, CR = .96, AVE = .65). The mother’s permissive style achieved acceptable internal consistency (α = .70) and CR (.81), with an AVE of .53, exceeding the recommended .50 cutoff (Fornell & Larcker, 1981), indicating adequate convergent validity (*Appendix* 1). Discriminant validity was confirmed using the Heterotrait-Monotrait ratio (HTMT), with all values remaining below the conservative threshold of .85, supporting the distinctiveness of the parenting style constructs (*Appendix* 2).

The Social Comparison Scale (Gibbons et al., 1999) was used to assess participants’ tendency to engage in social comparison. Th is 11 -item scale measures comparisons of abilities and orientations toward others’ opinions, with example items such as *“I always pay a lot of attention to how I do things compared with how others do things”* and *“I am not the type of person who compares myself often with others”* (reverse-coded). The original English version was translated into Vietnamese following a forward-backward translation procedure (Brislin, 1986). In this study, the scale demonstrated good internal reliability (α = .91, CR = .92) and adequate convergent validity (AVE = .52) (*Appendix 1*). HTMT values between social comparison and other constructs remained moderate, supporting discriminant validity (*Appendix 2*).

The Patient Health Questionnaire-4 (PHQ-4) (Kroenke et al., 2009) was used to assess participants’ psychological distress. This 4-item scale includes two items measuring anxiety and two items measuring depression over the past two weeks. Participants rated their responses on a 4-point Likert scale from 0 (Not at all) to 3 (Nearly every day), with higher scores indicating greater psychological distress. The Vietnamese version of this scale has been widely administered and exhibits high reliability and strong validity (Le et al., 2021; Nguyen et al., 2025; Tran et al., 2023; Tran et al., 2024). In this study, the PHQ-4 demonstrated high internal reliability (α = .87, CR = .91) and high convergent validity (AVE = .73) (*Appendix 1*). HTMT values between social comparison and other constructs remained moderate and supported discriminant validity (*Appendix 2*).

Academic performance was assessed using students’ most recent semester grade point average (GPA). A single-item question asked: *“What was your most recent semester GPA?”* The grading scale used was based on a 10-point system. Self-reported GPA was chosen because official academic records were not accessible for confidentiality reasons. Prior research indicates that self-reported GPA is highly correlated with official records (Kuncel et al., 2005; Shaw & Mattern, 2009).

### Statistical Analyses

We analyzed the data using SPSS 20.0, AMOS 20.0, and SmartPLS 4.0. First, we conducted Cronbach’s alpha analysis to examine the reliability of the scales. Internal consistency was examined using α and CR. Values exceeding .70 indicated acceptable reliability (Hair et al., 2013). Convergent validity was assessed using AVE, with the cutoff for AVE set at .50 (Fornell & Larcker, 1981) Discriminant validity was checked via the HTMT (< .85) ratio of correlations (Henseler et al., 2015). Next, we performed descriptive statistics, including mean, standard deviation, percentage distributions, and skewness/kurtosis for all study variables. Pearson correlation analysis was then conducted to explore the relationships among the study variables, with statistical significance set at p < .05. Finally, a multi-group SEM approach was employed using AMOS to examine the invariance of the structural model of mother’s parenting style across gender groups. The models for fathers and mothers were analyzed separately for male and female student groups. In each of the structural models, the independent variables consisted of parenting styles. Social comparison was specified as a mediating variable. The outcome variables included psychological distress and academic performance. The analysis followed a stepwise procedure including the configural, metric, scalar, and structural mean models. Model it was assessed using the Comparative Fit Index (CFI), the Root Mean Square Error of Approximation (RMSEA), and the chi-square divided by degrees of freedom (x^2^/df). Conventional thresholds were adopted, with CFI values above .95 and RMSEA values below .08 indicating acceptable fit, and a change in CFI (ACFI) less than .010 between nested models signifying model invariance (Cheung & Rensvold, 2002; Hair et al., 2013). To examine the hypothesized mediation efects, a bootstrapping procedure with 5.000 resamples was implemented at a 95% confidence interval. The indirect effect was considered signiicant if the conidence interval did not include zero.

## Results

*Table 1* summarizes the demographics and descriptive statistics for study variables by gender (N = 661). Th ere was no significant difference in age between males and females (t=-.99, *p* = .321, *d* = .08). For father’s authoritative style, scores were nearly identical for males and females (t = .08, *p* = .937, *d* = .01), whereas males scored significantly higher than females for father’s authoritarian style (t = 2.99, *p* = .003, *d* = .24). No significant gender difference was observed for father’s permissive style (t = .64, *p* = .523, *d* = .05). Regarding maternal parenting styles, females scored significantly higher in mother’s authoritative style compared to males (*t* = -2.09, *p* = .037, *d* = -.17), while males scored higher in mother’s authoritarian style (t = 2.23, *p* = .026, *d* = .18). There was no significant gender difference in mother’s permissive style (t = 1.00, *p* = .320, *d* = .08). For distress, the difference was not statistically significant between males and females (t=-1.19, *p* = .233, *d* = -.10), while females reported significantly higher social comparison scores than males (t = -2.16, *p* = .031, *d*=-.17). Finally, academic performance was significantly better in females compared to males (t = -2.54, *p* = .011, *d* = -.20).

**Table 1:** Demographics and Descriptive Statistics of Study Variables

Variable	Group	*Cohen’sd*	*Mean*	*SD*	*T*	*p-value*
Age	Male Female	.08	16.82 16.92	1.68 .96	-.99	.321
Father’s Authoritative Style	Male Female	.01	3.30 3.29	1.36 1.24	.08	.937
Father’s Authoritarian Style	Male Female	.24	2.46 2.20	1.24 1.01	2.99	.003
Father’s Permissive Style	Male Female	.05	2.59 2.54	1.21 .84	.64	.523
Mother’s Authoritative Style	Male Female	-.17	3.62 3.82	1.28 1.22	-2.09	.037
Mother’s Authoritarian Style	Male Female	.18	2.65 2.44	1.30 1.14	2.23	.026
Mother’s Permissive Style	Male Female	.08	2.73 2.65	1.22 .88	1.00	.320
Distress	Male Female	-.10	1.29 1.38	.93 .79	-1.19	.233
Social Comparison	Male Female	-.17	2.84 2.95	.70 .64	-2.16	.031
Academic Performance	Male Female	-.20	3.39 3.54	.79 .70	-2.54	.011

*Note. N = 661.*

*Table 2* shows the correlation between the research variables. Father’s parenting model invariance across gender was largely supported. *Tables 3 and 4* show the measurement invariance of the research models between males and females. For father’s parenting model, conigural and metric invariance were established, and partial scalar invariance was acceptable. Mother’s parenting model invariance across gender was supported. Conigural, partial metric, and partial scalar invariance were supported. Therefore, cross-group comparisons of latent means are considered acceptable.

**Table 2:** Correlation Matrix among Study Variables

Male (*N* = 246)									
Variable	1	2	3	4	5	6	7	8	9
1. Father’s Authoritative Style	1								
2. Father’s Authoritarian Style	49**	1							
3. Father’s Permissive Style	.59**	.79**	1						
4. Mother’s Authoritative Style	.68**	.35**	.43**	1					
5. Mother’s Authoritarian Style	.35**	.72**	.62**	.32**	1				
6. Mother’s Permissive Style	.42**	.68**	.68**	.46**	.80**	1			
7. Distress	.10	.31**	.31**	.04	.38**	.33**	1		
8. Social Comparison	.26**	.30**	.34**	.30**	.40**	.37**	.58**	1	
9. Academic Performance	.03	-.08	-.05	-.02	-.14*	-.08	-.04	.10	1
**Female** (N **= 415)**									
**Variable**	**1**	**2**	**3**	**4**	**5**	**6**	**7**	**8**	**9**
1. Father’s Authoritative Style	1								
2. Father’s Authoritarian Style	-.08	1							
3. Father’s Permissive Style	.29**	_.49**_	1						
4. Mother’s Authoritative Style	.59**	-.08	.19**	1					
5. Mother’s Authoritarian Style	-.19**	.60**	.36**	-.17**	1				
6. Mother’s Permissive Style	.04	.31**	.44**	.24**	.47**	1			
7. Distress	-.12*	.23**	.22**	-.13**	.35**	.22**	1		
8. Social Comparison	.01	.18**	.20**	.09	.20**	.17**	.39**	1	
9. Academic Performance	.11*	-.12*	.06	.08	-.07	.05	-.03	.16**	1

*Note. N = 661.*

**Table 3:** Gender Comparison of Father’s Parenting Model

Model	DF	CFI	RMSEA	ACFI	Conclusion
Unconstrained	0	1.000	-	-	Configural invariance achieved
Structural weights	14	.991	.029	ACFI = .009	Metric invariance achieved
Structural intercepts	17	.981	.038	ACFI = .010	Scalar invariance acceptable
Structural means	21	.970	.042	ACFI = .011	Partial scalar invariance recommended

**Table 4:** Gender Comparison of Mother’s Parenting Model

Model	DF	CFI	RMSEA	ACFI	Conclusion
Unconstrained	0	1.000	-	-	Conigural invariance achieved
Structural weights	14	.988	.034	ACFI = .012	Partial metric invariance recommended
Structural intercepts	17	.976	.043	ACFI = .012	Partial scalar invariance recommended
Structural means	21	.968	.045	ACFI = .008	Scalar invariance achieved

Analyses revealed distinct gender patterns in the efects of fathers’ parenting styles on adolescents’ outcomes (*Tables 5 and 6*). The authoritative style showed no signiicant indirect associations with academic performance through social comparison for either or females.

**Table 5:** Relationship Between Father’s Parenting Style and Academic Performance, Distress, with Social Comparison as a Mediator in Males

Paths	*β*	*p*	95% Confidence Interval	
			Lower	Upper
Direct effects				
Father’s Authoritative Style → Distress	-.18	.004	-.302	-.057
Father’s Permissive Style → Social Comparison	.23	.031	.020	.438
Social Comparison → Academic Performance	.13	.041	.006	.260
Social Comparison → Distress	.55	< .001	.441	.652
Indirect efects				
Father’s Permissive Style → Social Comparison → Distress	.13	.035	.009	.242
Total effects				
Father’s Permissive Style → Distress	.24	.027	.027	.446

*Note. R* ^*2*^ *: Academic Performance = .09, Distress = .38, Social Comparison = .12. Only statistically significant paths are reported; see Appendix 3 for more details.*

**Table 6:** Relationship Between Father’s Parenting Style and Academic Performance, Distress, with Social Comparison as a Mediator in Females

Paths	*β*	*P*	95% Confidence Interval	
			Lower	Upper
Direct effects				
Father’s Authoritarian Style → Academic Performance	-.13	<.001	-.209	-.058
Father’s Authoritative Style → Distress	-.10	<.001	-.156	-.040
Father’s Permissive Style → Distress	.15	.002	.055	.253
Father’s Permissive Style → Social Comparison	.12	.008	.030	.206
Social Comparison → Academic Performance	.18	<.001	.081	.287
Social Comparison → Distress	.44	<.001	.328	.542
Indirect efects				
Father’s Permissive Style → Social Comparison → Academic Performance	.02	.035	.002	.042
Father’s Permissive Style → Social Comparison → Distress	.05	.012	.011	.092
Total efects				
Father’s Authoritarian Style → Academic Performance	-.12	.002	-.198	-.045
Father’s Permissive Style → Academic Performance	.10	.035	.007	.198
Father’s Authoritative Style → Distress	-.11	<.001	-.168	-.043
Father’s Authoritarian Style → Distress	.09	.037	.005	.174
Father’s Permissive Style → Distress	.21	<.001	.101	.311

*Note. R^2^: Academic Performance = .11; Distress = .23; Social Comparison =. 05. Only statistically significant paths are reported; see Appendix 4 for more details.*

The authoritarian style predicted lower academic performance (Β = -.13, *p* = .002) and higher distress (Β = .09, *p* = .037) among females, but effects were nonsignificant for males. The permissive style displayed the strongest associations. For females, it was positively related to academic performance both directly (Β = .10, *p* = .035) and indirectly via social comparison (*Β* = .02, *p* = .035). For both genders, permissive fathering predicted higher distress through social comparison (males: Β = .13, *p* = .035; females: Β = .05, *p* = .012). Model explanatory power was moderate, with higher R^2^ for males (social comparison = .12; psychological distress = .38) than females (.05 and .23, respectively). Overall, social comparison emerged as a signiicant mediating pathway linking paternal style to adolescent mental health.

Analyses of mothers’ parenting styles revealed clear gender-specific patterns in both direct and indirect effects (*Tables 7 and 8*). Higher levels of authoritative mothers’ parenting predicted better academic performance through social comparison (Β = .03, *p* = .049) and greater distress via the same pathway among males (Β = .10, *p* = .008). Th ese effects were not significant for females. Although total effects on academic performance were nonsigniicant across genders, the direct efect on distress was significant for females (Β = -.09, *p* = .002). For authoritarian parenting, sig-niicant indirect efects were observed for both genders on academic performance (males: Β = .06, *p* = .033; females: Β = .02, *p* = .017) and distress (males: Β = .17, *p* = .002; females: Β = .05, *p* = .002). Total effects showed that this style increased distress in both groups (males: Β = .31, *p* = .002; females: Β = .16, *p* < .001). In contrast, permissive parenting showed no signiicant direct or indirect efects on any outcome for either gender. The models accounted for more variance among males (R^2^ = .11 for academic performance; .40 for distress; .20 for social comparison) than among females (.09, .27, and .06, respectively).

**Table 7:** Relationship Between Mother’s Parenting Style and Academic Performance, Distress, with Social Comparison as a Mediator in Males

Paths	*β*	*P*	95% Confidence Interval	
			Lower	Upper
Direct Effects				
Mother’s Authoritarian Style → Academic Performance	-.25	.015	-.453	-.048
Mother’s Authoritative Style → Distress	-.22	<.001	-.334	-.111
Social Comparison → Academic Performance	.19	.005	.058	.321
Social Comparison → Distress	.55	<.001	.442	.658
Indirect Efects				
Mother’s Authoritative Style → Social Comparison → Academic Performance	.03	.J	.001	.067
Mother’s Authoritarian Style → Social Comparison → Academic Performance	.06	.033	.005	.113
Mother’s Authoritative Style → Social Comparison → Distress	.10	.008	.025	.170
Mother’s Authoritarian Style → Social Comparison—> Distress	.17	.002	.062	.280
Total Efects				
*#x2003;Mother’s Authoritarian Style → Distress	.31	.002	.113	.499

*Note. R^2^: Academic Performance = .11; Distress = .40; Social Comparison = .2. Only statistically significant paths are reported; see Appendix 5 for more details.*

**Table 8:** Relationship Between Mother’s Parenting Style and Academic Performance, Distress, with Social Comparison as a Mediator in Females

Paths	*β*	*P*	95% Confidence Interval	
			Lower	Upper
Direct Effects				
Mother’s Authoritarian Style → Academic Performance	-.10	.008	-.164	-.025
Mother’s Authoritative Style → Distress	-.09	.002	-.151	-.034
Mother’s Authoritarian Style → Distress	.16	<.001	.087	.226
Social Comparison → Academic Performance	.18	<.001	.077	.285
Social Comparison → Distress	.43	<.001	.328	.538
Indirect Efects				
Mother’s Authoritarian Style → Social Comparison → Academic Performance	.02	.017	.003	.035
Mother’s Authoritarian Style → Social Comparison → Distress	.05	.002	.017	.076
Total Efects				
Mother’s Authoritarian Style → Academic Performance	-.08	.034	-.145	-.006
Mother’s Authoritative Style → Distress	-.07	.030	-.131	-.007
Mother’s Authoritarian Style → Distress	.20	<.001	.129	.277

*Note. R^2^: Academic Performance = .09. Distress = .27. Social Comparison = .06. Only statistically significant paths are reported; see Appendix 6 for more details.*

## Discussion

Comparing the total efects between fathers and mothers reveals distinct gender patterns. For fathers, neither authoritative nor authoritarian styles showed signii-cant associations with boys’ academic performance, although authoritarian style was negatively linked to girls’ outcomes. Permissive fathering was positively related to girls’ academic performance but associated with increased distress in both genders. Regarding distress, authoritative and authoritarian styles were not signiicantly related to boys’ outcomes, but were for girls. In contrast, for mothers, neither authoritative nor permissive styles were signiicantly associated with academic performance, while the authoritarian style showed stronger negative links for girls. In terms of distress, only the mother’s authoritative style was associated with reduced distress in girls, whereas the authoritarian style was related to greater distress across both genders.

This result can be explained through a cultural lens. In Vietnamese society, fathers are traditionally seen as the primary disciplinarians and authority igures within the family (Düng, 2007). This cultural dynamic might help explain why the father’s authoritarian style negatively impacted girls’ academic performance but did not have the same efect on boys. In Vietnam, girls are ot en expected to conform to strict behavioral norms, and this can create higher levels of stress when faced with authoritarian parenting (Do Minh, 2024; Hiên, 2023). Vietnamese daughters are frequently raised to be more obedient, disciplined, and academically focused (Hiên, 2023) than sons, which means they may experience greater emotional strain when their fathers adopt authoritarian approaches that emphasize control over emotional warmth. This inding aligns with other research that has shown that girls may be more sensitive to authoritarian parenting (Mehr-un-Nisa Idrees et al., 2021; Rakhshani et al., 2022). For boys, however, the authoritarian style of parenting might align more closely with societal expectations for masculinity, which ot en stress independence and emotional restraint (An et al., 2022; Nguyen, 2024). Therefore, boys may not react as strongly to authoritarian control and could even view it as reinforcing the expectations placed on them as male children.

The father’s permissive style was positively associated with girls’ academic performance, but showed no significant association for boys. This outcome may be attributed to the greater emphasis on emotional nurturing in Vietnam, which girls may be more receptive to. In Vietnamese cultural contexts, social expectations have traditionally encouraged boys to develop independence and self-reliance, whereas girls have ot en been guided toward cultivating emotional sensitivity and interpersonal connectedness (Düng, 2007; Linh, 2019). Th ese patterns do not necessarily apply uniformly, but reflect broader socialization tendencies. Within such a framework, a permissive parenting style might better resonate with children, particularly girls, who beneit from emotional support and encouragement in developing their socio-emotional skills. Boys may respond differently to a permissive parenting style, as cultural expectations in many Vietnamese families tend to emphasize autonomy and emotional restraint in boys’ upbringing. The more relaxed, nurturing approach of permissive parenting may thus not align with the traditional gender expectations for boys, who may be perceived as needing less emotional support and more discipline.

The mother’s role in Vietnamese families is typically centered around emotional support and caregiving, with mothers ot en seen as the nurturers who manage the emotional and social well-being of the children (Hiên, 2023; Linh, 2019). Th is traditional view of mothers is likely a contributing factor to the indings of our study regarding the mother’s authoritative style, which was found to signiicantly reduce distress in girls but not in boys. In Vietnam, girls are ot en raised to be more emotionally expressive and relational, and they may thus respond more positively to the emotional guidance and empathy provided by mothers. The authoritative style may align well with the cultural expectation that mothers provide emotional security and support (Anh, 2023; Linh, 2019). Girls, therefore, may experience a reduction in distress when their mothers engage in authoritative parenting that fosters emotional understanding and autonomy. For boys, however, the efects of authoritative parenting may be less. In a culture where boys are ot en encouraged to suppress emotions and focus on independence (Vu, 2021), the emotional support provided by authoritative parenting may not resonate as strongly with them. Thus, while authoritative parenting may be beneicial for reducing distress in girls, it does not seem to have the same impact on boys, relecting the gendered emotional expectations prevalent in Vietnamese society.

Additionally, mothers’ authoritarian style was associated with higher levels of distress in both boys and girls, with a stronger association observed in girls. This could be explained by the highly demanding and controlling nature of authoritarian parenting, which does not allow for emotional expression or autonomy (Yafe, 2023). In Vietnamese culture, girls are oten expected to be more obedient and responsive to parental authority than boys (Dang, 2023; Quy, 2024). Therefore, the lack of emotional warmth and the strict control typical of authoritarian parenting can create sig-niicant distress for girls, who may feel pressured to meet high expectations without receiving suicient emotional support (Dang, 2023). For boys, the link between authoritarian parenting and distress appeared weaker than for girls, which may relect gendered expectations that discourage emotional expression in response to control. However, the authoritarian approach is still harmful in both cases, as it stiles emotional growth and the ability to self-regulate, which are important for both genders (Yildiz & Altay, 2021). Moreover, in Vietnamese society, academic success is highly valued, especially for children in urban areas where there is signiicant pressure to perform well in school. This pressure can amplify the effects of parenting styles, particularly authoritarian parenting, which may lead to heightened anxiety and distress for children who already feel overwhelmed by academic expectations.

### Father’s Parenting Style and Social Comparison

An especially notable aspect of these indings is the role of social comparison as a mediator, with distinct patterns emerging based on parenting style and child gender. For boys, the association between authoritarian parenting and distress appeared weaker, possibly reflecting gendered social norms that discourage emotional expression in response to parental control. This is particularly noteworthy in the Vietnamese cultural context, where fathers traditionally serve as authority igures, prioritizing discipline over emotional closeness (An et al., 2022; Düng, 2007; Linh, 2019). Authoritative parenting may not substantially alter children’s social comparison tendencies, especially in a culture where emotional bonds with fathers are typically less emphasized than those with mothers. Both authoritative and authoritarian fathers may establish clear behavioral standards for their children, reducing the need for them to compare themselves with peers. When parents set explicit expectations, children may rely on these predeined standards rather than external comparisons to guide their behavior (Festinger, 1957; Suls & Wills, 2024). Consistent with this perspective, our i ndings indicate that the father’s authoritarian style also showed no signiicant indirect efects, suggesting that direct control or leniency from fathers may not inluence children’s academic performance or emotional well-being through social comparison.

The father’s permissive style was positively associated with academic performance through social comparison in females, but this indirect efect was not signii-cant in males. This suggests that daughters may be more influenced by social comparison when their fathers are overly indulgent or neglectful in academic matters. Although the father’s permissive style was associated with better academic outcomes in females, it was also positively linked to increased distress through social comparison in both genders. These findings suggest that permissive parenting may have complex efects, potentially beneiting academic performance while contributing to higher psychological distress. The permissive style may create an emotional space that encourages social comparison (Festinger, 1957). Without clear behavioral guidelines from their parents, children may rely on external comparisons to establish self-evaluation standards (Swathi et al., 2024). This effect could be particularly relevant in Vietnamese society, where daughters oten face strict societal expectations regarding academic performance and personal conduct (Quy, 2024; Vân, 2014). This may make them more susceptible to the pressures of social comparison.

Regarding distress, the father’s permissive style was associated with signiicant indirect relationships through social comparison for both genders, with a relatively stronger association observed in males. In Vietnamese culture, permissiveness can create confusion or emotional strain in children, as they may struggle with self-regulation in the absence of strict parental guidance. This can lead to heightened distress, particularly in an environment where academic pressure is intense (Pham & Pham, 2021). The significant indirect effect on both males and females suggests that while permissive parenting ofers emotional support, it may lack the necessary structure to help children manage academic demands, potentially contributing to greater distress. Without clear parental expectations, children may ind it diicult to assess their own abilities and behaviors. According to social comparison theory, this uncertainty can drive them to compare themselves with others to establish personal standards (Festinger, 1957; Suls & Wills, 2024). Moreover, in Vietnam’s collectivist society, the lack of parental guidance may have even more negative efects. Even if children do not necessarily feel pressured to compete academically, they may still engage in social comparison to identify shared behavioral norms within their peer groups. This tendency could lead to distress, especially if they adopt unrealistic standards, reinforcing feelings of inadequacy or frustration.

### Mother’s Parenting Style and Social Comparison

For the mother’s authoritative style, the indirect efect on academic performance via social comparison was signiicant for males but not for females. In Vietnamese culture, mothers are typically seen as the primary emotional caregivers (Hiên, 2023; Vân, 2014), and their approach to parenting can significantly influence social comparison, particularly when it involves seeking approval or recognition. Males, who may experience pressure to meet the high academic expectations placed on them, could be more likely to engage in social comparison in response to maternal encouragement and guidance. This could drive them to improve their academic performance to meet both internal and external standards. On the other hand, females may not engage in social comparison in response to maternal support in the same way, possibly due to diferent emotional expectations or societal pressures that emphasize obedience over competitiveness (Vân, 2014).

The mother’s authoritarian style had significant indirect effects on both academic performance and distress through social comparison for both genders. Strict control and high expectations in parenting can encourage children to compare themselves with others, especially in eastern countries (Tian et al., 2017). Academic success remains a top priority in Vietnamese culture, leading authoritarian mothers to set rigid expectations. Boys and girls may feel pressured to evaluate their progress against peers, which can enhance academic performance but also increase emotional strain. High standards from mothers can create stress and anxiety in children who struggle to meet these expectations. Girls appeared more afected, possibly due to additional societal pressures on academic achievement and social behavior. Maternal strictness, combined with cultural norms, can intensify social comparison, amplifying stress and emotional distress.

## Theoretical Implications

This study makes important contributions to developmental psychology by deepening the understanding of the associations among parenting styles, social comparison, academic performance, and distress within the Vietnamese cultural context. The indings highlight gender diferences in how parenting styles relate to academic and psychological outcomes through the mechanism of social comparison. Speciically, the father’s permissive style shows a distinct pattern of associations: it corresponds with higher academic performance scores in females, while being linked to increased distress levels in both males and females. This style of parenting may be accompanied by less explicit behavioral guidelines or expectations, which can relate to greater uncertainty for adolescents. Such uncertainty may be associated with a heightened tendency toward social comparison, particularly in a cultural environment like Vietnam where gender roles and expectations remain strongly inluential.

Our results suggest that mothers play an important role in children’s academic and psychological distress within the Vietnamese cultural context. This study contributes to the literature by highlighting associations between the mother’s authoritarian style and both academic performance and distress through social comparison across genders. In a Vietnamese cultural context, where academic success is emphasized and strict parenting is more common, authoritarian mothers may inadvertently create a high-stakes environment that encourages social comparison, potentially leading to greater academic achievement but also higher distress levels in children. This contributes to the growing body of literature that suggests authoritarian styles may exacerbate emotional distress while enhancing academic drive. In Vietnam, where mothers often take on the role of emotional support and guidance, their authoritative approach may encourage males to compare themselves with peers more actively, which could lead to better academic performance. This difference in response could be rooted in cultural expectations that emphasize achievement and success for boys, while girls are oten expected to be more compliant and obedient, which might limit the inluence of authoritative parenting on their social comparisons.

This study advances social comparison theory (Festinger, 1957) by establishing social comparison as a core psychological determinant of adolescent mental health and academic outcomes in Vietnam. The findings reveal that social comparison exerts a stronger and more direct efect on psychological distress than any parenting-related factor. The results align with the expanding scholarship on comparison culture within East Asian education systems, where individual worth is closely bound to academic achievement and relative ranking. In Vietnam, this dynamic is intensiied by familial expectations, competitive schooling, and achievement-oriented socialization (Lôc et al., 2024; Pham & Pham, 2021). Within this sociocultural framework, social comparison becomes an important driver that organizes how adolescents perceive success and self-worth. These insights require a reconceptualization of adolescent developmental models in Vietnam, positioning achievement pressure and comparison-based self-evaluation as primary, not contextual variables in understanding adolescent mental health. The study further extends the theory by illuminating gender-specific mechanisms in social comparison. The data indicate that comparison processes are more intensely activated among boys, particularly under maternal authoritarian influence. This gendered pattern highlights the necessity for Social Comparison Theory (Festinger, 1957) to explicitly integrate cultural and gender dimensions when explaining how individuals internalize evaluative hierarchies in collectivist, achievement-driven societies.

## Practical Implications

Our study provides several practical insights for parents, educators, and policymakers in Vietnam, particularly concerning the associations between parenting styles, social comparison, and students’ academic performance and psychological distress. By recognizing the differential effects of fathers’ and mothers’ parenting approaches, interventions can be tailored to reduce academic distress while promoting healthy academic motivation.

First, given that the father’s permissive style is associated with higher academic performance in females through social comparison, while also being related to increased distress for both genders, parents should recognize that excessive leniency and lack of structure may not be as beneficial as they appear. The absence of clear guidance can inadvertently cause children to lose direction in their learning and to base their self-worth on external evaluations rather than intrinsic competence. This is particularly relevant in the Vietnamese cultural context, where academic success often serves as a primary indicator of personal value and family reputation. Th ere-fore, parents should help adolescents develop the capacity to regulate their emotions and strive for achievement grounded in personal growth rather than in relative performance. Listening to emotions and providing constructive feedback focused on personal growth and efort can bring many beneits to children’s psychological development (Kennedy, 2024).

Second, since the mother’s authoritarian style is associated with higher academic performance as well as increased distress through social comparison, schools and mental health professionals should consider the potential psychological challenges linked to strict parenting. Vietnamese mothers may unintentionally reinforce a competitive and high-pressure academic environment that increases stress and self-doubt in children. Parental education programs can help mothers adopt more balanced strategies, such as promoting self-improvement over competition and fostering internal motivation rather than external comparisons. Interventions must guide mothers to de-escalate overly controlling behaviors and, critically, to eliminate comparative and conditional language. They should replace phrases like “why can’t you be more like the neighbor’s child?” with language that validates effort and autonomy. The goal is to dismantle the source of external pressure that forces children into an unhealthy social comparison pattern for self-validation.

Third, the mother’s authoritative style was associated with higher academic performance through social comparison in males, suggesting that open communication and encouragement may be linked to boys’ academic motivation. Schools and counseling programs should encourage mothers to foster open dialogue with their sons, helping them use social comparison in a constructive manner rather than as a source of self-doubt (Huynh et al., 2024; Oppenheim & Koren-Karie, 2014). To achieve this, schools and counseling programs can implement workshops that focus on open emotional communication and perspective-taking in daily life. These sessions should include role-playing and relective exercises that help parents and children recognize both the positive and negative aspects of social comparison. Furthermore, parents and educators can play an active role in helping adolescents engage in constructive forms of social comparison. They can initiate open conversations about efort, persistence, and self-development to redirect students’ focus from competition toward learning and self-improvement. Schools can integrate social-emotional learning programs that teach students to notice moments of social comparison in academic or peer contexts, to label the emotions that arise, and to analyze the thoughts that accompany these feelings. Through guided reflection and structured classroom activities, students can learn how to interpret comparison as information for self-improvement instead of as a judgment of personal worth. The programs can also include exercises in goal setting and self-monitoring so that adolescents translate comparison experiences into speciic, attainable objectives that enhance motivation and emotional balance. Additionally, guided group relections or peer-mentoring programs can provide safe spaces where adolescents learn to interpret diferences as sources of inspiration and mutual learning rather than as threats to self-worth. These combined strategies may help adolescents build emotional resilience, maintain intrinsic motivation, and use social comparison as a positive force for academic and psychological development.

## Limitations

This study has several limitations that should be considered. One key limitation is that the study does not distinguish between upward and downward social comparison. Th ese two types of social comparison may have different effects on academic performance and distress. The present study focused on ability and opinion social comparison, but did not include measures of upward or downward comparison. Future research should investigate the distinct roles of upward and downward social comparison in academic and psychological outcomes by modeling them separately in mediation analyses. Dual mediation models may clarify whether each type of comparison differentially mediates the effects of specific parenting styles.

One more notable limitation lies in the measurement properties of the father’s permissive parenting subscales (α = .66). Th ese figures suggest potential measurement inconsistency within the father’s permissive parenting construct in the Vietnamese cultural context.

Another limitation concerns the measurement of parenting style. Parenting style was assessed solely based on the child’s self-report, which may not fully reflect parents’ actual behaviors. Although children’s perceptions of parenting are theoretically and empirically important, future research should include parental self-reports or observational measures to obtain a more comprehensive view of parenting practices.

Longitudinal studies are strongly recommended to establish temporal and causal relationships between parenting styles, social comparison tendencies, and student outcomes. Cross-lagged panel designs could help identify reciprocal inluences over time. In addition, incorporating qualitative approaches could offer deeper insight into students’ subjective experiences of social comparison and how they perceive parental inluence. Expanding the research across diverse cultural contexts would also allow for cross-cultural comparisons and enhance the generalizability of inding.

Finally, this study relied on self-reported GPA as a measure of academic performance. Although prior meta-analyses have shown strong correlations between self-reported and official GPA (Kuncel et al., 2005), the possibility of minor recall errors or social desirability bias cannot be ruled out. Future research could verify academic performance using institutional records to enhance measurement accuracy.

## Appendix Appendix 1

**Table:** Average Variance Extracted and Reliability Coefficients of the Measurement Scales

Variables/Items	Factor Loading	A	CR (rho_c)	AVE
Father’s Authoritative Style		.96	.96	.67
F_AS_01	.80			
F_AS_02	.80			
F_AS_03	.80			
F_AS_04	.82			
F_AS_05	.84			
F_AS_06	.84			
F_AS_07	.88			
F_AS_08	.82			
F_AS_09	.82			
F_AS_10	.84			
F_AS_11	.71			
F_AS_12	.83			
F_AS_13	.80			
Father’s Authoritarian Style		.94	.95	.60
F_AU_01	.53			
F_AU_02	.73			
F_AU_03	.77			
F_AU_04	.84			
F_AU_05	.81			
F_AU_06	.86			
F_AU_07	.83			
F_AU_08	.69			
F_AU_09	.85			
F_AU_10	.84			
F_AU_11	.83			
F_AU_12	.75			
F_AU_13	.68			
Father’s Permissive Style		.66	.80	.50
F_PS_01	.44			
F_PS_02	.80			
F_PS_03	.89			
F_PS_04	.30			
Mother’s Authoritative Style		.96	.96	.66
M_AS_01	.79			
M_AS_02	.82			
M_AS_03	.82			
M_AS_04	.84			
M_AS_05	.84			
M_AS_06	.80			
M_AS_07	.85			
M_AS_08	.79			
M_AS_09	.81			
M_AS_10	.85			
M_AS_11	.73			
M_AS_12	.82			
M_AS_13	.81			
Mother’s Authoritarian Style		.95	.96	.65
M_AU_01	.56			
M_AU_02	.81			
M_AU_03	.82			
M_AU_04	.82			
M_AU_05	.83			
M_AU_06	.88			
M_AU_07	.86			
M_AU_08	.73			
M_AU_09	.87			
M_AU_10	.86			
M_AU_11	.86			
M_AU_12	.79			
M_AU_13	.74			
Mother’s Permissive Style		.70	.81	.53
M_PS_01	.84			
M_PS_02	.75			
M_PS_03	.44			
M_PS_04	.81			
Psychological Distress		.87	.91	.73
PD_01	.87			
PD_02	.87			
PD_03	.80			
PD_04	.86			
Social Comparison		.91	.92	.52
SC_01	.73			
SC_02	.72			
SC_03	.80			
SC_04	.75			
SC_05	.51			
SC_06	.78			
SC_07	.72			
SC_08	.78			
SC_09	.77			
SC_10	.75			
SC_11	.59			

## Appendix 2

**Table:** Heterotrait-Monotrait Ratios Between Measurement Scales

	1	2	3	4	5	6	7	8
1. Father’s Authoritative Style								
2. Father’s Authoritarian Style	.15							
3. Father’s Permissive Style	.33	.76						
4. Mother’s Authoritative Style	.65	.09	.15					
5. Mother’s Authoritarian Style	.05	.69	.59	.09				
6. Mother’s Permissive Style	.18	.55	.79	.24	.66			
7. Psychological Distress	.19	.28	.40	.22	.33	.41		
8. Social Comparison	.03	.28	.33	.05	.39	.23	.56	

## Appendix 3

**Table:** Relationship Between Father’s Parenting Style and Academic Performance, Distress, with Social Comparison as a Mediator in Males (Table 5)

Paths	Beta	*P*	95% Conidence Interval	
			Lower	Upper
Direct efects				
Father’s Authoritative Style → Academic Performance	.09	.219	-.056	.242
Father’s Authoritarian Style → Academic Performance	-.14	.158	-.338	.055
Father’s Permissive Style → Academic Performance	-.03	.772	-.246	.183
Father’s Authoritative Style → Distress	-.18	.004	-.302	-.057
Father’s Authoritarian Style → Distress	.15	.073	-.014	.310
Father’s Permissive Style → Distress	.11	.218	-.066	.288
Father’s Authoritative Style → Social Comparison	.09	.216	-.054	.237
Father’s Authoritarian Style → Social Comparison	.07	.461	-.120	.265
Father’s Permissive Style → Social Comparison	.23	.031	.020	.438
Social Comparison → Academic Performance	.13	.041	.006	.260
Social Comparison → Distress	.55	< .001	.441	.652
Indirect efects				
Father’s Authoritative Style → Social Comparison → Academic Performance	.01	.290	-.010	.035
Father’s Authoritarian Style → Social Comparison → Academic Performance	.01	.488	-.018	.037
Father’s Permissive Style → Social Comparison → Academic Performance	.03	.138	-.010	.071
Father’s Authoritative Style → Social Comparison → Distress	.05	.219	-.030	.130
Father’s Authoritarian Style → Social Comparison → Distress	.04	.463	-.066	.145
Father’s Permissive Style → Social Comparison → Distress	.13	.035	.009	.242
Total efects				
Father’s Authoritative Style → Academic Performance	.11	.167	-.044	.255
Father’s Authoritarian Style → Academic Performance	-.13	.191	-.330	.066
Father’s Permissive Style → Academic Performance	.00	.991	-.216	.213
Father’s Authoritative Style → Distress	-.13	.082	-.275	.016
Father’s Authoritarian Style → Distress	.19	.057	-.005	.381
Father’s Permissive Style → Distress	.24	.027	.027	.446

*Note. Academic Performance = .09, Distress = .38, Social Comparison =. 12.*

## Appendix 4

**Table:** Relationship Between Father’s Parenting Style and Academic Performance, Distress, with Social Comparison as a Mediator in Females (Table 6)

Paths	Estimate	^**p**^	95% Conidence Interval	
			Lower	Upper
Direct efects				
Father’s Authoritative Style → Academic Performance	.04	.200	-.019	.092
Father’s Authoritarian Style → Academic Performance	-.13	<.001	-.209	-.058
Father’s Permissive Style → Academic Performance	.08	.095	-.014	.176
Father’s Authoritative Style → Distress	-.10	<.001	-.156	-.040
Father’s Authoritarian Style → Distress	.06	.125	-.017	.141
Father’s Permissive Style → Distress	.15	.002	.055	.253
Father’s Authoritative Style → Social Comparison	-.02	.530	-.069	.035
Father’s Authoritarian Style → Social Comparison	.07	.073	-.006	.135
Father’s Permissive Style → Social Comparison	.12	.008	.030	.206
Social Comparison → Academic Performance	.18	<.001	.081	.287
Social Comparison → Distress	.44	<.001	.328	.542
Indirect efects				
Father’s Authoritative Style → Social Comparison → Academic Performance	.00	.537	-.013	.007
Father’s Authoritarian Style → Social Comparison → Academic Performance	.01	.110	-.003	.026
Father’s Permissive Style → Social Comparison → Academic Performance	.02	.035	.002	.042
Father’s Authoritative Style → Social Comparison → Distress	-.01	.531	-.030	.015
Father’s Authoritarian Style → Social Comparison → Distress	.03	.080	-.003	.060
Father’s Permissive Style → Social Comparison → Distress	.05	.012	.011	.092
Total efects				
Father’s Authoritative Style → Academic Performance	.03	.247	-.023	.090
Father’s Authoritarian Style → Academic Performance	-.12	.002	-.198	-.045
Father’s Permissive Style → Academic Performance	.10	.035	.007	.198
Father’s Authoritative Style → Distress	-.11	<.001	-.168	-.043
Father’s Authoritarian Style → Distress	.09	.037	.005	.174
Father’s Permissive Style → Distress	.21	<.001	.101	.311

*Note. Academic Performance = .11; Distress = .23; Social Comparison =. 05*

## Appendix 5

**Table:** Relationship Between Mother’s Parenting Style and Academic Performance, Distress, with Social Comparison as a Mediator in Males (Table 7)

Paths	Estimate	P	95% Conidence Interval	
			Lower	Upper
Direct Efects				
Mother’s Authoritative Style → Academic Performance	-.01	.917	-.143	.128
Mother’s Authoritarian Style → Academic Performance	-.25	.015	-.453	-.048
Mother’s Permissive Style → Academic Performance	.05	.652	-.163	.260
Mother’s Authoritative Style → Distress	-.22	<.001	-.334	-.111
Mother’s Authoritarian Style → Distress	.13	.113	-.032	.300
Mother’s Permissive Style → Distress	.12	.189	-.057	.290
Social Comparison → Academic Performance	.19	.005	.058	.321
Social Comparison → Distress	.55	<.001	.442	.658
Indirect Efects				
Mother’s Authoritative Style → Social Comparison → Academic Performance	.03	.049	.001	.067
Mother’s Authoritarian Style → Social Comparison → Academic Performance	.06	.033	.005	.113
Mother’s Permissive Style → Social Comparison → Academic Performance	.01	.679	-.030	.047
Mother’s Authoritative Style → Social Comparison → Distress	.10	.008	.025	.170
Mother’s Authoritarian Style → Social Comparison → Distress	.17	.002	.062	.280
Mother’s Permissive Style → Social Comparison → Distress	.02	.676	-.087	.134
Total Efects				
Mother’s Authoritative Style → Academic Performance	.03	.703	-.109	.162
Mother’s Authoritarian Style → Academic Performance	-.19	.062	-.393	.010
Mother’s Permissive Style → Academic Performance	.06	.604	-.158	.272
Mother’s Authoritative Style → Distress	-.13	.060	-.255	.005
Mother’s Authoritarian Style → Distress	.31	.002	.113	.499
Mother’s Permissive Style → Distress	.14	.183	-.066	.346

*Note. R^2^.: Academic Performance = .11; Distress = .40; Social Comparison = .20*

## Appendix 6

**Table:** Relationship Between Mother’s Parenting Style and Academic Performance, Distress, with Social Comparison as a Mediator in Females (Table 8)

Paths	Estimate	P	95% Conidence Interval	
			Lower	Upper
Direct Efects				
Mother’s Authoritative Style → Academic Performance	.01	.705	-.047	.069
Mother’s Authoritarian Style → Academic Performance	-.10	.008	-.164	-.025
Mother’s Permissive Style → Academic Performance	.08	.098	-.014	.165
Mother’s Authoritative Style → Distress	-.09	.002	-.151	-.034
Mother’s Authoritarian Style → Distress	.16	<.001	.087	.226
Mother’s Permissive Style → Distress	.07	.120	-.019	.161
Social Comparison → Academic Performance	.18	<.001	.077	.285
Social Comparison → Distress	.43	<.001	.328	.538
Indirect Efects				
Mother’s Authoritative Style → Social Comparison → Academic Performance	.01	.086	-.001	.021
Mother’s Authoritarian Style → Social Comparison → Academic Performance	.02	.017	.003	.035
Mother’s Permissive Style → Social Comparison → Academic Performance	.01	.329	-.008	.023
Mother’s Authoritative Style → Social Comparison → Distress	.02	.053	-.003	.047
Mother’s Authoritarian Style → Social Comparison → Distress	.05	.002	.017	.076
Mother’s Permissive Style → Social Comparison → Distress	.02	.312	-.017	.055
Total Efects				
Mother’s Authoritative Style → Academic Performance	.02	.482	-.037	.079
Mother’s Authoritarian Style → Academic Performance	-.08	.034	-.145	-.006
Mother’s Permissive Style → Academic Performance	.08	.071	-.007	.174
Mother’s Authoritative Style → Distress	-.07	.030	-.131	-.007
Mother’s Authoritarian Style → Distress	.20	<.001	.129	.277
Mother’s Permissive Style → Distress	.09	.069	-.007	.187

*Note. R2.- Academic Performance = .09. Distress = .27. Social Comparison = .06*

## References

[ref1] Agbaria, Q., Mahamid, F., & Veronese, G. (2021). The Association between attachment patterns and parenting styles with emotion regulation among Palestinian preschoolers. Sage Open, 11(1), 2158244021989624. 10.1177/2158244021989624

[ref2] An, T.L., Waling, A., & Bourne, A. (2022). Men and masculinities studies in Vietnam: A brief review. Sociology Compass, 16(3), e12965. 10.1111/soc4.12965

[ref3] Anh, N.H. (2023). Vai tro cüa ngüÖi phu nü trong gin giü giá tri gia dinh truyên thông và xây düng gia dinh the dô thai ky moi [The role of women in preserving traditional family values and building modern families in the capital city]. Tap chíkhoa hoc ViêtNam trUc tuyên [Vietnam Science Journal Online], (1), 21–33.

[ref4] Azman, Ö., Mauz, E., Reitzle, M., Geene, R., Holling, H., & Rattay, P. (2021). Associations between parenting style and mental health in children and adolescents aged 11-17 years: Results of the KiGGS Cohort Study (second follow-up). Children, 8(8), 672. 10.3390/children8080672 34438563 PMC8394813

[ref5] Barberis, N., Cannavo, M., Cuzzocrea, F., & Verrastro, V. (2022). Alexithymia in a Self Determination Theory framework: The interplay of psychological basic needs, parental autonomy support and psychological control. Journal of Child and Family Studies, 32. 10.1007/s10826-022-02303-3

[ref6] Bibi, A., Hayat, R., Hayat, N., Zulfiqar, S., Shafique, N., & Khalid, M.A. (2022). Impact of parenting styles on psychological flexibility among adolescents of Pakistan: A cross-sectional study. Child and Adolescent Social Work Journal, 39(3), 313–322. 10.1007/s10560-021-00754-z

[ref7] Braza, P., Carreras, R., Muñoz, J.M., Braza, F., Azurmendi, A., Pascual-Sagastizábal, E., Cardas, J., & Sánchez-Martín, J.R. (2015). Negative maternal and paternal parenting styles as predictors of children’s behavioral problems: Moderating effects of the child’s sex. Journal of Child and Family Studies, 24(4), 847–856. 10.1007/s10826-013-9893-0

[ref8] Brislin, R.W. (1986). The wording and translation of research instruments. In W. J. Lonner & J. W. Berry (Eds.), Field methods in cross-cultural psychology (pp. 137–164). Sage Publications.

[ref9] Buri, J.R. (1991). Parental Authority Questionnaire. Journal of Personality Assessment, 57(1), 110–119. 10.1207/s15327752jpa5701_13 16370893

[ref10] Cheung, G.W., & Rensvold, R.B. (2002). Evaluating goodness-of-it indexes for testing measurement invariance. Structural Equation Modeling, 9(2), 233–255. 10.1207/S15328007SEM0902_5

[ref11] Chung, T., & Mallery, P. (1999). Social comparison, individualism-collectivism, and self-esteem in China and the United States. Current Psychology, 18(4), 340–352. 10.1007/s12144-999-1008-0

[ref12] Costa, S., Sireno, S., Larcan, R., & Cuzzocrea, F. (2019). The six dimensions of parenting and adolescent psychological adjustment: The mediating role of psychological needs. Scandinavian Journal of Psychology, 60(2), 128–137. 10.1111/sjop.12507 30556135

[ref13] Dang, D.K. (2023). Families and problems of Vietnamese families in the process of international integration. VNUHCM Journal of Social Sciences and Humanities, 7(S1), S34–S41. https://doi. org/10.32508/stdjssh.v7iS1.922

[ref100] Dang, T.K.á., & Nguyen, T.N. (2025). Nhân thúc cüa sinh viên vê sü üng hô cüa cha me dôi vói tính tü chü cüa con: nghiên cúu tai môt sô trüÖng dai hoc d Thành phô Hô Chí Minh [Students’ perceptions of parental support for their autonomy: A study at selected universities in Ho Chi Minh City]. Tap chí Giáo duc [Journal of Education], 25(7), 47–52. https://tcgd.tapchigiaoduc.edu.vn/index. php/tapchi/article/view/3119

[ref101] Devina, A., & Hendrawan, D. (2023). The mediating role of parent autonomy support in the relationship of parenting stress and 4-6 years old child’s social competence. AIP Conference Proceedings, 2679(1). 10.1063/5.0111296

[ref102] Dô Minh, T. (2024). Tü tüdng hô chí minh vê sü cân thiêt phâi giâi phóng phu nü d Viêt Nam [Ho Chi Minh’s thought on the necessity of women’s liberation in Vietnam]. Tap chí Khoa hoc xä hôi Thành phô Hô Chí Minh [Journal of Social Sciences of Ho Chi Minh City], 8(314), 1–14. http://tapchikhx-hhcm.org.vn/index.php/tapchikhxh/article/view/400

[ref103] Düng, B.Q. (2007). Gia dinh trong các xä hôi nông nghiêp [The family in agrarian societies]. Tap chíXä hôi hoc [Journal of Sociology], 103–112.

[ref104] Festinger, L. (1957). Social Comparison Theory. Journal of Selective Exposure Theory, 16(401), 3.

[ref105] Fornell, C., & Larcker, D.F. (1981). Evaluating structural equation models with unobservable variables and measurement error. Journal of Marketing Research, 18(1), 39–50. 10.2307/3151312

[ref106] Gawas, A.G.A. (2021). Parenting styles, social responsibility and their relationship to academic achievement among Yemeni high school students in Turkey. International Journal of Social and Humanities Sciences Research (JSHSR), 8(70), 1307–1315. 10.26450/jshsr.2430

[ref107] Giang, T.V., Huynh, V.S., Sâm, V.L., & Lê, N.K. (2023). Phân tích däc diêm tâm lí cûa hành vi toan tu sát d vi thành niên: nghiên cúu môt sô trüÖng hÖp tai khu vüc thành phô hô chí minh [An Analysis of the psychological characteristics of suicidal behavior among adolescents: A case study in Ho Chi Minh City]. Tap chí Khoa hoc Tnldng Dai hoc Sil pham TP Hô Chí Minh [Journal of Science, Ho Chi Minh City University of Education], 20(4), 579. 10.54607/hcmue.js.20.4.3787(2023)

[ref108] Gibbons, F.X., & Buunk, A. (1999). Individual differences in social comparison: Development of a scale of social comparison orientation. Journal of Personality and Social Psychology, 76(1), 129–142. 10.1037/0022-3514.76.1.129 9972558

[ref109] Gilbert, P., McEwan, K., Bellew, R., Mills, A., & Gale, C. (2009). The dark side of competition: How competitive behaviour and striving to avoid inferiority are linked to depression, anxiety, stress and self-harm. Psychology and Psychotherapy: Theory, Research and Practice, 82(2), 123–136. 10.1348/147608308X379806 19040794

[ref110] Goagoses N., Bolz T., Eilts J., Schipper N., Schütz J., Rademacher A., Vesterling C., Koglin U. (2023). Parenting dimensions/styles and emotion dysregulation in childhood and adolescence: A systematic review and Meta-analysis. Current Psychology, 42(22), 18798–18822. 10.1007/s12144-022-03037-7

[ref111] Gorostiaga, A., Aliri, J., Balluerka, N., & Lameirinhas, J. (2019). Parenting styles and internalizing symptoms in adolescence: A systematic literature review. Int. J. Environ. Res. Public Health, 16(17), 3192. 10.3390/ijerph16173192 31480548 PMC6747480

[ref112] Guo, X., & Slesnick, N. (2018). The mediating role of autonomy and relatedness on maternal and child outcomes. Journal of Abnormal Child Psychology, 46(2), 209–221. 10.1007/s10802-017-0303-8 28474188

[ref113] Guyer, A.E., Caouette, J.D., Lee, C.C., & Ruiz, S.K. (2014). Will they like me? Adolescents’ emotional responses to peer evaluation. International Journal of Behavioral Development, 38(2), 155–163. 10.1177/0165025413515627 25076803 PMC4112521

[ref114] Hair, J.F., Black, W.C., Babin, B.J., & Anderson, R.E. (2013). Multivariate data analysis. Pearson Education Limited. https://books.google.com.vn/books?id=VvXZnQEACAAJ

[ref115] Hassan, M., Malik, A. S., Sang, G., Rizwan, M., Mushtaque, I., & Naveed, S. (2022). Examine the parenting style effect on the academic achievement orientation of secondary school students: The moderating role of digital literacy [Original Research]. Frontiers in Psychology, 13, 1063682. 10.3389/fpsyg.2022.1063682 36591109 PMC9798310

[ref116] Henseler, J., Ringle, C.M., & Sarstedt, M. (2015). A new criterion for assessing discriminant validity in variance-based structural equation modeling. Journal of the Academy of Marketing Science, 43(1), 115–135. 10.1007/s11747-014-0403-8

[ref117] Hiên, P. T. T. (2023). Van hoá gia dinh Viêt Nam thai phong kiên và nhüng giá tri kê thiïa trong bôi cânh xây düng nhà nüóc pháp quyên xä hôi chü nghïa [Vietnamese family culture during the feudal period and its inherited values in the context of building a socialist rule-of-law state]. Tap chí Khoa hoc Xä hôi Viêt Nam [Vietnam Journal of Social Sciences], *11,* 71-78.

[ref118] Hock, R.S., Mendelson, T., Surkan, P. J., Bass, J.K., Bradshaw, C.P., & Hindin, M.J. (2018). Parenting styles and emerging adult depressive symptoms in Cebu, the Philippines. Transcultural Psychiatry, 55(2), 242–260. 10.1177/1363461517748813 29493429

[ref119] Huynh, H.P., Thomas, J., Castellanos, I., Weatherford, D.R., & Lilley, M.K. (2024). Social comparison, be-longingness, self-doubt, and stress: The case of Hispanic students at Hispanic majority institutions. Hispanic Journal of Behavioral Sciences, 46(2), 99–121. 10.1177/07399863241292243

[ref120] Kennedy, K. (2024). Authoritative parenting and the impact on child development.

[ref121] Kroenke, K., Spitzer, R.L., Williams, J.B., & LÖwe, B. (2009). An ultra-brief screening scale for anxiety and depression: the PHQ-4. Psychosomatics, 50(6), 613–621. 10.1176/appi.psy.50.6.613 19996233

[ref122] Kuncel, N.R., Credé, M., & Thomas, L.L. (2005). The validity of self-reported grade point averages, class ranks, and test scores: A meta-analysis and review of the literature. Review of Educational Research, 75(1), 63–82. 10.3102/00346543075001063

[ref123] La, T.T.T., Dinh, H.V.T., Phan, M.H.T., Do, L.H.T., Nguyen, P.H.T., & Nguyen, Q.A.N. (2020). Mental health among Vietnamese urban late adolescents: The association of parenting styles. Health Psychology Open, 7(2), 2055102920948738. 10.1177/2055102920948738 PMC8851146 35186310

[ref124] Le. H.N.T., Le, M.-A.T., Nguyen, H.T., Vo, H.-V., Le, N.Q., Tang, L.N.P., Tran, T.T., & Le, T.V. (2021). Patient Health Questionnaire (PHQ-9): A depression screening tool for people with epilepsy in Vietnam. Epilepsy & Behavior, 125, 108446. 10.1016/j.yebeh.2021.108446 34839244

[ref125] Linh, T.T.D. (2019). Giáo duc cüa ngüÖi cha dOi vol con trong gia dmh hat nhân Ö viêt nam hiên nay [Fathers’ education of their children in nuclear families in contemporary vietnam]. TNU Journal of Science Technology, 209(16), 34–41.

[ref126] Liu, H., Kvintova, J., & Vachova, L. (2025). Parents’ social comparisons and adolescent self-esteem: the mediating effect of upward social comparison and the moderating influence of optimism. Frontiers in Psychology, 16, 1473318. 10.3389/fpsyg.2025.1473318 PMC11794513 39911989

[ref127] Lôc, T.H.V., Vân Anh, H.N., & Kiên, T.G. (2024). Trâm câm, lo âu, cäng thâng và các yêu tô liên quan Ö hoc sinh trung hoc phÖ thông tai Thành phô Hô Chi Minh [Depression, anxiety, stress, and related factors among high school students in Ho Chi Minh City].

[ref128] Mai, M.H. (2024). Các yêu tô ânh hüÖng dên hành vi tu hüy hoai bân thân cüa vi thành niên tai các dô thi phia nam, Viêt Nam [Factors affecting self-destructive behavior among adolescents in southern urban areas of Vietnam]. Tap chí Khoa hoc Tnldng Dai hoc Sil pham TP Hô Chi Minh [Journal of Science, Ho Chi Minh City University of Education], 21(1), 71. 10.54607/hcmue.js.21.1.3932(2024)

[ref129] Mehr-un-Nisa Idrees, S.M., Zahra, F.N., & Naeem, N. (2021). Perceived parenting styles and primary attachment styles of single and children living with both parents. J. Pak. Med. Assoc., 71, 1540. 10.47391/JPMA.626 34111068

[ref130] Milevsky, A., Schlechter, M., Netter, S., & Keehn, D. (2007). Maternal and paternal parenting styles in adolescents: Associations with self-esteem, depression and life-satisfaction. Journal of Child and Family Studies, 16(1), 39–47. 10.1007/s10826-006-9066-5

[ref131] Nguyen, H., Nguyen, H.T., Nguyen, N.B., Tran, D., Harvey, D.J., Nguyen, B.T., Nguyen, B.T., Nguyen, A.N., Nguyen, C.T.H., Nguyen, T.T. H., Nguyen, T.L., Nguyen, A.T.P., Nguyen, N.H., Nguyen, A.L., Luong, Y.H., Nguyen, B.H., Nguyen, P.Q., Gitlin, L.N., Nguyen, T.A., ... Hinton, L. (2025). Testing the efficacy of a culturally adapted family dementia caregiver intervention (REACH VN): Results from a cluster randomized controlled trial in northern Vietnam. The American Journal of Geriatric Psychiatry, 33(5), 535–545. 10.1016/j.jagp.2024.10.011 PMC11903202 39547823

[ref132] Nguyen, N. (2024). Masculine norms, mental health stigma, and help-seeking among men in vietnam: a mixed methods study.

[ref133] Nguyêt, N.T.M. (2010). Hành vi bao lut cüa cha me dôi vói con tuÖi vi thành niên. In: TrüOng Dai hoc Khoa hoc Xâ hôi và Nhân Vän [Parental violent behavior toward adolescent children. In: University of Social Sciences and Humanitie], Luán vän thac si ngành Tâm ly hoc [Master’s Thesis in Psychology].

[ref134] Oppenheim, D., & Koren-Karie, N. (2014). Parental insightfulness and child-parent emotion dialogues: Their importance for children’s development. In Mechanisms of social connection: From brain to group. (pp. 205–220). American Psychological Association. 10.1037/14250-012

[ref135] Peng, B., Hu, N., Yu, H., Xiao, H., & Luo, J. (2021). Parenting style and adolescent mental health: The chain mediating effects of self-esteem and psychological inflexibility. Frontiers in psychology, 12, 738–170. 10.3389/fpsyg.2021.738170 PMC8548717 34721210

[ref136] Pham, T.P.T., & Pham, T.H.T. (2021). áp lUc hoc tap Ö hoc sinh trung hoc phÖ thông: Vai tro và trách nhiêm cüa cha me [Academic pressure among high school students: The role and responsibility of parents]. Tap chí Giáo duc [Journal of Education], 1–6.

[ref137] Pinquart, M. (2016). Associations of parenting styles and dimensions with academic achievement in children and adolescents: A meta-analysis. Educational Psychology Review, 28(3), 475–493. https:// doi.org/10.1007/s10648-015-9338-y

[ref138] Quach, A.S., Epstein, N.B., Riley, P.J., Falconier, M.K., & Fang, X. (2015). Effects of parental warmth and academic pressure on anxiety and depression symptoms in Chinese adolescents. Journal of Child and Family Studies, 24(1), 106–116. 10.1007/s10826-013-9818-y

[ref139] Quy, M.T. (2024). Thüc trang giáo duc giá tri cho nü sinh viên mot sô trüdng dai hoc, cao dâng d thanh hóa hiên nay [The current situation of value education for female students at some universities and colleges in Thanh Hoa]. Tap cht Khoa hoc Triidng Daihoc Hong Dúc [Hong Duc University Journal of Science], 53.

[ref140] Rakhshani, T., Hamid, S., Kamyab, A., Kashfi, S.M., & Jeihooni, A.K.J.H. (2022). The effect of parenting style on anxiety and depression in adolescent girls aged 12-16 years. 8(11), e11478. https://doi. org/10.1016/j.heliyon.2022.e11478 10.1016/j.heliyon.2022.e11478 PMC9663855 36387519

[ref141] Romero-Acosta, K., Gómez-de-Regil, L., Lowe, G.A., Lipps, G.E., & Gibson, R.C. (2021). Parenting styles, anxiety and depressive symptoms in child/adolescent. Int J Psychol Res (Medellin), 14(1), 12–32. 10.21500/20112084.4704 34306576 PMC8297574

[ref142] Ryan, R.M., & Deci, E.L. (2000). Self-determination theory and the facilitation of intrinsic motivation, social development, and well-being. American Psychologist, 55(1), 68–78. 10.1037/0003-066X.55.1.68 11392867

[ref143] Shaw, E.J., & Mattern, K.D. (2009). Examining the Accuracy of Self-Reported High School Grade Point Average. Research Report No. 2009-5. College Board.

[ref144] Stavrulaki, E., Li, M., & Gupta, J. (2021). Perceived parenting styles, academic achievement, and life satisfaction of college students: The mediating role of motivation orientation. European Journal of Psychology of Education, 36(3), 693–717. 10.1007/s10212-020-00493-2

[ref145] Suls, J., & Wills, T.A. (2024). Social comparison: Contemporary theory and research. Taylor & Francis.

[ref146] Swathi, P., Dhamecha, J., & Bharmal, K. (2024). Parenting styles, emotional regulation and social comparison among young adults. In Health Psychology in Integrative Health Care (pp. 247–258). Rout-ledge. 10.4324/9781003596806-36

[ref147] Szkody, E., Steele, E.H., & McKinney, C. (2021). Effects of parenting styles on psychological problems by self esteem and gender differences. 42(9), 1931–1954. 10.1177/0192513x20958445

[ref148] Thao, A.T. (2025). Parenting styles among Among Fathers: Associations with children’s grades and learning behaviors. Keiser University.

[ref149] Tian, L., Yu, T., & Huebner, E.S. (2017). Achievement goal orientations and adolescents’ subjective well-being in school: The mediating roles of academic social comparison directions. Frontiers in psychology, 8, 37. 10.3389/fpsyg.2017.00037 28197109 PMC5281619

[ref150] Timpano, K.R., Carbonella, J.Y., Keough, M.E., Abramowitz, J., & Schmidt, N.B. (2015). Anxiety sensitivity: An examination of the relationship with authoritarian, authoritative, and permissive parental styles. J Cogn Psychother, 29(2), 95–105. 10.1891/0889-8391.29.2.95 32759160

[ref151] Tran, B., Nguyen, M., Auquier, P., Boyer, L., Fond, G., Vu, G.T., Hoang, T.P., Ho, P.T., Nguyen, T.H., Latkin, C.A., Ho, C.S., Ho, R.C.M., & Zhang, M. (2023). Psychological impacts of COVID-19 on Vietnamese health workers over the prolonged restricted COVID-19 responses: A cross-sectional study. BMJ Open, 13, e069239. 10.1136/bmjopen-2022-069239 PMC10401210 37536968

[ref152] Tran, V.D., Vo, T.M.L., Vo, Q.L.D., Nguyen, M.T., Nguyen, M.C., Dewey, R.S., & Nguyen, T.H.Y. (2024). Behavioral factors associated with medication adherence among hypertensive patients using the theoretical domains framework. Exploratory Research in Clinical and Social Pharmacy, 16, 100510. 10.1016/j.rcsop.2024.100510 39399764 PMC11470627

[ref153] Vân, T.N. (2014). Ihuyët tam tong, tú dúc trong nho giáo và ánh hiidng cúa nó dói vói phu nü Vibèt Nam hiên nay [The doctrine of Three Obediences and Four Virtues in Confucianism and its influence on Vietnamese women today]. Hoc Viên Chính Tri Quóc Gia Hô Chi Minh [Ho Chi Minh National Academy of Politics].

[ref154] Vu, T.T. (2021). Love, affection and intimacy in marriage of young people in Vietnam. Asian Studies Review, 45(1), 100–116. 10.1080/10357823.2020.1798873

[ref155] Watabe, A., & Hibbard, D.R. (2014). The influence of authoritarian and authoritative parenting on children’s academic achievement motivation: A comparison between the United States and Japan. North American Journal of Psychology, 16(2), 359–382.

[ref156] Wilder, S. (2014). Effects of parental involvement on academic achievement: a meta-synthesis. Educational Review, 66(3), 377–397. 10.1080/00131911.2013.780009

[ref157] Yaffe, Y. (2023). Systematic review of the differences between mothers and fathers in parenting styles and practices. Current Psychology, 42(19), 16011–16024. 10.1007/s12144-020-01014-6

[ref158] Yang, J., & Zhao, X. (2020). Parenting styles and children’s academic performance: Evidence from middle schools in China. Children and Youth Services Review, 113, 105–017. 10.1016/j.childyouth.2020.105017

[ref159] Yeon, E., & Choi, H. (2022). The relationship between parenting style, perceived academic achievement pressure on elementary school students’ school adjustment and life satisfaction: Focused on the mediating effect of autonomy and academic stress. Korean Association for Learner-Centered Curriculum and Instruction, 22, 313–328. 10.22251/jlcci.2022.22.14.313

[ref160] Yildiz, S., & Altay, N. (2021). The parenting attitudes and effects on their gifted children: A literature review. Journal for the Education of Gifted Young Scientists, 9(2), 123–132. 10.17478/jegys.864037

[ref161] Yu, Y., & Ho, H.-Z. (2023). The influence of Taiwanese parenting style on adolescent mental health and academic performance. International Journal about Parents in Education, 12, 85–95. 10.54195/ijpe.14115

